# The human neonatal small intestine has the potential for arginine synthesis; developmental changes in the expression of arginine-synthesizing and -catabolizing enzymes

**DOI:** 10.1186/1471-213X-8-107

**Published:** 2008-11-10

**Authors:** Eleonore S Köhler, Selvakumari Sankaranarayanan, Christa J van Ginneken, Paul van Dijk, Jacqueline LM Vermeulen, Jan M Ruijter, Wouter H Lamers, Elisabeth Bruder

**Affiliations:** 1 Department of Anatomy & Embryology, Maastricht University, Maastricht, The Netherlands; 2 Department of Veterinary Medicine, Veterinary Anatomy & Embryology, University of Antwerp, Belgium; 3 AMC Liver Center Academic Medical Center, University of Amsterdam, The Netherlands; 4 Department of Anatomy & Embryology, Academic Medical Center, University of Amsterdam, The Netherlands; 5 Department of Pathology, Basel University Hospital, Basel, Switzerland

## Abstract

**Background:**

Milk contains too little arginine for normal growth, but its precursors proline and glutamine are abundant; the small intestine of rodents and piglets produces arginine from proline during the suckling period; and parenterally fed premature human neonates frequently suffer from hypoargininemia. These findings raise the question whether the neonatal human small intestine also expresses the enzymes that enable the synthesis of arginine from proline and/or glutamine. *Carbamoylphosphate synthetase (CPS), ornithine aminotransferase (OAT), argininosuccinate synthetase (ASS), arginase-1 (ARG1), arginase-2 (ARG2), and nitric-oxide synthase (NOS) were visualized by semiquantitative immunohistochemistry in 89 small-intestinal specimens*.

**Results:**

Between 23 weeks of gestation and 3 years after birth, CPS- and ASS-protein content in enterocytes was high and then declined to reach adult levels at 5 years. OAT levels declined more gradually, whereas ARG-1 was not expressed. ARG-2 expression increased neonatally to adult levels. Neurons in the enteric plexus strongly expressed ASS, OAT, NOS1 and ARG2, while varicose nerve fibers in the circular layer of the muscularis propria stained for ASS and NOS1 only. The endothelium of small arterioles expressed ASS and NOS3, while their smooth-muscle layer expressed OAT and ARG2.

**Conclusion:**

The human small intestine acquires the potential to produce arginine well before fetuses become viable outside the uterus. The perinatal human intestine therefore resembles that of rodents and pigs. Enteral ASS behaves as a typical suckling enzyme because its expression all but disappears in the putative weaning period of human infants.

## Background

Arginine is a precursor for the synthesis of proteins, creatine, agmatine, and nitric oxide (NO). It further plays an essential role in ammonia and bicarbonate detoxification, and stimulates the secretion of growth hormone, prolactin, insulin, and glucagon. Arginine is also a 'conditionally essential' amino acid, meaning that endogenous arginine production covers metabolic requirements in healthy, unstressed individuals, but becomes an essential amino acid under conditions of increased need, e.g. growth or tissue repair, or in catabolic states such as sepsis and starvation.

**Figure 1:**
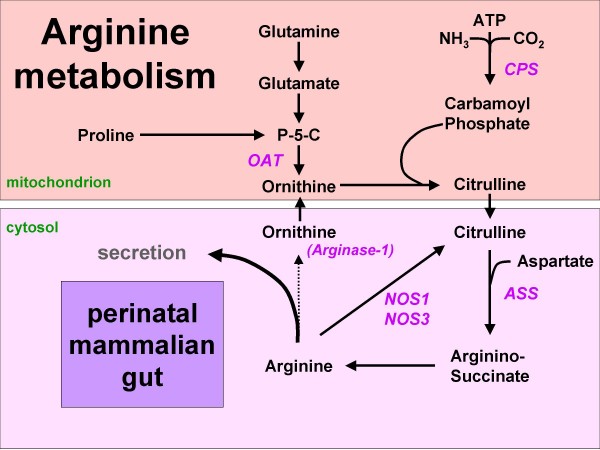
**Arginine synthesis from proline or glutamine in the mammalian neonatal gut**. Since arginase-1 is not expressed, arginine can either be secreted or metabolized to NO and citrulline. Names of enzymes investigated in this study are indicated in italics. P-5-C = pyrroline5-carboxylate synthetase.

In the adult, endogenous arginine biosynthesis is an inter-organ 'affair': the net production of citrulline occurs almost exclusively in the enterocytes of the small intestine [1], also in man [2], but absorption of citrulline from the circulation and subsequent biosynthesis of arginine can take place in many tissues [3]. Of these, the cortex of the kidney provides approximately 20% of whole-body requirements [4]. In perinatal mice [5,6] and piglets [7-9], however, all enzymes necessary for arginine biosynthesis from proline and glutamine (Figure 1) are expressed in the enterocytes of the small intestine, while ARG1, the main cytosolic arginine-catabolizing enzyme, is not detectable prior to weaning [5,6,10]. In agreement, the small intestine plays a prominent role in net arginine production in suckling piglets [11-14]. In rodents, intestinal expression of the enzymes that synthesize arginine from citrulline, ASS and argininosuccinate lyase, ceases completely after weaning [6,15]. In pigs, on the other hand, net synthesis of arginine declines more gradually and is still present at 7 weeks of age [16]. It has been speculated that enteric arginine synthesis is necessary to cover neonatal requirements, because mammalian milk is a relatively poor source of arginine, whereas its precursors proline and glutamine are abundant [17].

In prematurely born human neonates, hypoargininemia is frequently observed [18] and hypothesized to predispose such infants to the development of necrotizing enterocolitis [19-21]. Although hypoargininemia in premature human neonates has been associated with failing intestinal arginine biosynthesis as found in suckling rodents and piglets [22], no evidence to support this association exists thus far. To confirm or reject the hypothesis that the perinatal human gastrointestinal tract resembles that of rodents or pigs with respect to arginine production, we studied the developmental changes in the expression of CPS, OAT and ASS, three key enzymes with a high control of de novo intestinal synthesis of citrulline and arginine, and ARG1 and ARG2, the main arginine-catabolizing enzymes in full-thickness and mucosal biopsies of the human small intestine. The findings demonstrate that the epithelium of the fetal and neonatal small intestine abundantly expresses CPS (as we reported earlier [23,24]), OAT and ASS, whereas cytosolic ARG1 is not detectable. These data show that the perinatal human intestine resembles that of rodents and, in particular, pigs with respect to its capacity to produce arginine. We also show that the expression of the controlling enzyme, ASS, all but disappears between 3 and 5 years of age, that is, the putative weaning age of human infants [25]. Finally, we show that the enteric ganglia and arteriolar endothelium co-express ASS and the constitutive NO-synthases NOS1 and NOS3, respectively, which both use arginine as substrate for NO synthesis.

## Methods

### Tissue

A total of 89 samples were included in the study (Table 1). Formalin-fixed, paraffin-embedded samples originated from the archives of the Institute of Pathology, University Hospital Basel, Switzerland, and the Department of Pathology, AMC, Amsterdam, the Netherlands. Full-thickness duodenal, jejunal and ileal samples were from infants, who presented with gastroschisis, atresia, meconium ileus or Meckel's diverticulum, whereas the samples from adults were from patients who underwent surgery for tumors. Duodenal mucosal biopsies were collected from patients who underwent endoscopy for various gastrointestinal complaints. Furthermore, full-thickness intestinal samples of 9 fetuses between 14 and 40 gestational weeks were examined. For each sample, age, gender and diagnosis were available, but in all other respects, the samples had been anonymized to avoid patient identification. Under this condition, residual tissue could be used for the research reported here [26]. In addition, approval has been obtained from the Ethikkommission beider Basel EKbB, Switzerland, reference number EK 135/08 for the samples obtained from the Institute of Pathology in Basel.

**Table 1:** Human gut samples

**Group**	**Age**	**# of samples**	**Sample origin**				**Resections**	**Biopsies**
			***SI***	***D***	***J***	***I***		
1	-182/-3 days	9	7		2		9	0
2	1 – 11 days	14	1	4	3	6	14	0
3	6 wks – 1 yr	10	4	1		5	9	1
4	1,5 – 3 yrs	12	2	7		3	5	7
5	3 – 5 yrs	16*		15		3	0	16
6	5 – 7 yrs	18*		16		5	2	16
7	14 – 22 yrs	5		1		4	4	1
8	50 – 80 yrs	5		4		1	5	0

The table shows the number of samples that were analyzed per age group, the topographic location of the samples (SI = small intestine, D = duodenum, J = jejunum, I = ileum), and whether they were mucosal biopsies and full-thickness resections. For age groups 5 and 6, parallel samples were available from two different regions of the small intestine. These were used to check for the effect of location on enzyme expression, but only one sample per patient was taken into account for the statistical analysis. This is the reason for the discrepancy between "# of samples" (marked by an asterisk) and the sum of all samples listed under "Sample origin".

### Immunohistochemistry

Tissue was fixed in 4% formaldehyde and embedded in paraffin following standard protocols. 5–7 μm-thick sections were used. Section thickness does not affect immunohistological staining intensity, since antibodies only bind to the surface of the sections [27]. As an additional cautionary measure to avoid staining differences due to differences in fixation, we used neuronal staining as an internal reference. Sections were deparaffinized, hydrated in graded ethanols, and heated in 10 mM sodium citrate (pH 6.0) to 98°C for 10 minutes followed by 90 minutes cool-down to room-temperature to retrieve antigens. After this treatment, endogenous alkaline phosphatases are denatured and no longer active. Remaining activity of endogenous peroxidases (catalase) was inactivated where appropriate by exposing the sections for 30 min to 3% H_2_O_2 _in PBS. After blocking with TENG-T (10 mM Tris pH 7.4, 5 mM EDTA, 150 mM NaCl, 0.25% gelatin, 0.05% Tween-20) plus 10% goat serum, sections were incubated overnight at room temperature with the first antibody, washed in 0.5 M Na-acetate and incubated with an alkaline phosphatase- or peroxidase-coupled secondary antibody for 60 to 90 minutes. Sections were developed with NBT/BCIP (alkaline phosphatase; Roche) or DAB (peroxidase; Sigma). An incubation without primary antibody served as negative control for all incubations [see Additional file 1]. This protocol allows semi-quantitative assessment on sections [28].

The following rabbit primary antibodies were used: ASS (1:10,000) [15]; ARG1 and ARG2 (1:500, sc-20150 and 1:400, sc-20151, respectively, Santa Cruz Biotechnology, California); CPS (1:500) [29]; OAT (1:1,500) [30]. Antibody binding was visualized with alkaline phosphatase-coupled goat anti-rabbit IgG (1:200, Sigma A-3687). NOS1 and NOS3 were detected with mouse monoclonal antibodies (NOS1 1:400, IgG2a clone #16; NOS3 1:200, IgG1 clone #3, BD Transduction Laboratories) and visualized with an alkaline phosphatase-coupled goat anti-mouse secondary antibody (1:200, Sigma A3562) and a peroxidase-coupled rabbit anti-mouse secondary antibody (1:200, Sigma A3682), respectively. Single NOS1 positive cells in the lamina propria were further characterized by staining for the presence of CD68 (monocytes/macrophages 1:200, Dako M0876), CD3 (T-lymphocytes 1:600, Dako A0452), CD20 (B-cells 1:500, Dako M0755 clone L26), CD1A (dendritic cells 1:10, Neomarkers MS-1856-P1), and CD138 (plasma cells 1:100, Dako M7228 clone MI15).

### Western Blotting

Villus epithelium was scraped off fresh duodenal resection material and lysed in SDS-PAGE sample buffer. After separation of samples on 10% SDS-polyacrylamide gels and blotting to PVDF membranes, proteins were visualized with antisera to ARG1 (1:200); ARG2 (1:500); NOS1 (1:2500); NOS3 (1:2500); α-SMA, (1:1000). The appropriate secondary horse-radish peroxidase-coupled antibodies were used at a dilution of 1:10,000. The signal was amplified using the chemiluminescent Super Signal West Pico reagent (Pierce, Perbio Science, The Netherlands) and pictures were taken with a LAS3000 imaging system (Fujifilm). α-smooth muscle actin was used to determine the contribution of the submucosa to the scrapings.

### Evaluation of samples

Sections were scored for staining intensity in random sequence by 3 investigators with the readers of the slides blinded. Villi and crypts were scored separately. The intensity of ASS, OAT and ARG2 staining in enterocytes was expressed on a scale of 0–3 (0: absent; 1: weak; 2: intermediate; and 3: strong expression) relative to their expression in the ganglia of the myenteric plexus, which always contained both strongly and weakly positive neurons for ASS, OAT, and ARG2. Expression in the strongly staining neurons was set at 3. Samples without ganglia (mucosal biopsies from the duodenum) were compared to simultaneously stained samples that did contain ganglia to assign an intensity score from 0 to 3. Because CPS expression in the small intestine is restricted to enterocytes, all samples were stained simultaneously and then compared to each other using the same scale of 0–3 as described above. There was never more than one scoring unit difference between the 3 investigators.

### Statistics

We used box-plots to visualize distribution and developmental changes of enzyme expression in enterocytes. Two-way analysis of variance (ANOVA) on rank-transformed data showed age-group and structure (crypt or villus) differences as well as age and structure interactions per enzyme. Sex (male or female) was not a factor associated with differences in enzyme expression. To pin-point which age-groups and crypts or villi differed, non-parametric one-way ANOVA (Kruskal-Wallis) tests were performed between age-groups per structure. Multiple comparison (Mann-Whitney) tests between structures per age-group were performed, when the null hypothesis was rejected. P values were considered significant if < 0.05.

## Results

The earliest sample studied was a small intestine of a 14-week-old fetus, whereas the oldest sample was from a 79 year-old patient. Most of the samples examined were from patients younger than 7 years. At 14 weeks of gestation, all structural components of the epithelium, enteric nerves, and smooth muscle layers have formed [31,32]. Specimens were from the duodenum, jejunum, or ileum, with the majority of samples originating from the duodenum and ileum (Table 1). From 4 patients (age groups 5 and 6; 3–7 years), parallel samples of duodenum and ileum were available. In these samples, no differences in staining intensities of the enzymes investigated were observed between the proximal and distal small intestine or between males and females. For these 2 reasons, we felt justified to pool the samples of one age group. Of necessity, the studied specimens included both mucosal biopsies and full-thickness specimens, were all obtained to diagnose gastrointestinal conditions, and were contributed by different institutions. Because the gastrointestinal conditions were diverse in nature and the observed changes in enzyme levels concordant, our conclusions reflect developmental biology rather than pathology. Based on the staining patterns in these samples, we describe the developmental changes in the expression of arginine-synthesizing enzymes CPS, ASS and OAT, and the arginine-metabolizing enzymes ARG1, ARG2, NOS1 and NOS3 in the enterocytes of the small intestine.

### Expression of CPS, ASS, OAT, ARG, NOS1 and NOS3 in enterocytes

#### Carbamoylphosphate synthetase (Figures 2A and 3; [see also Additional files 2 and 3])

**Figure 2:**
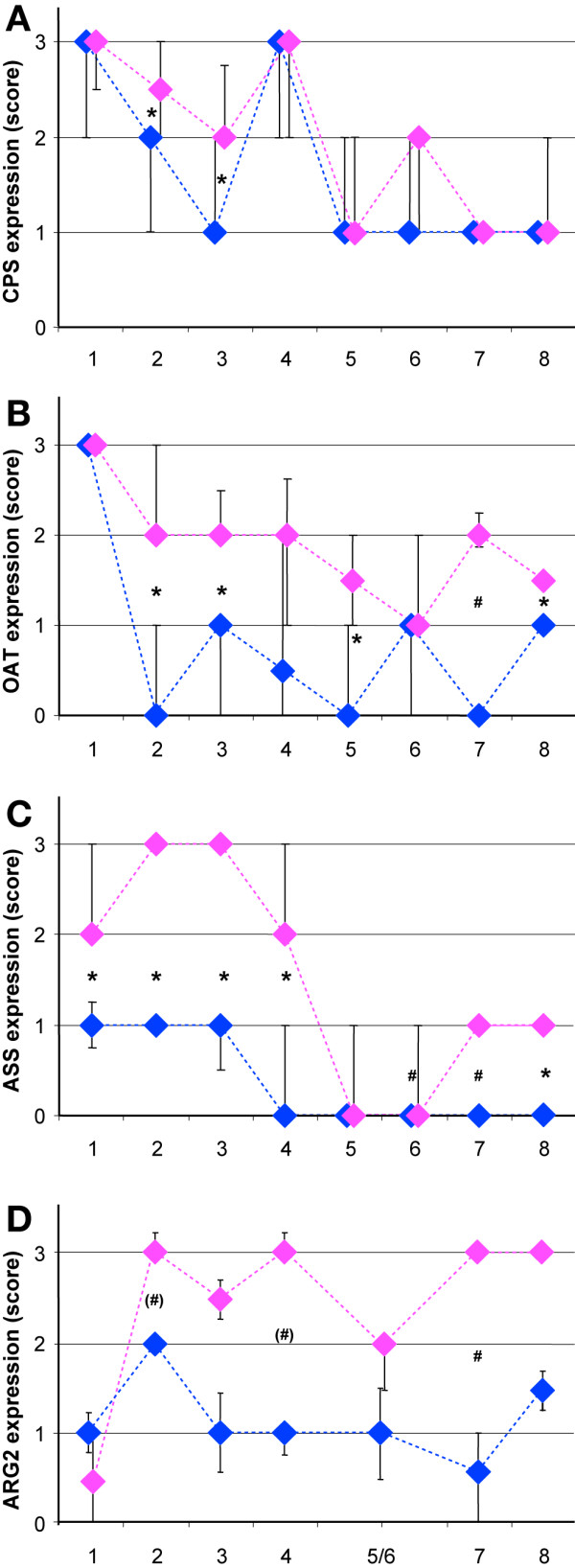
**Developmental changes in expression of CPS, ASS, OAT and ARG2 in enterocytes of the small intestine**. Sections represented in the top three panels (CPS, ASS and OAT) were stained simultaneously, whereas a subset of the samples with 2–4 individual sections per age group was stained later and is represented in the bottom panel (ARG2). The staining intensities were graded on a scale of 0 to 3. Medians of all observations of each age-group are indicated by diamonds and are plotted for villi (pink) and crypts (blue) separately. The vertical bars represent the first and third quartiles of each age group. When not drawn, the quartile coincides with the median. Age groups are: 1: fetus (14^th ^– 39^th ^week of pregnancy); 2: 1–11 postnatal days; 3: 42–365 days; 4: 1.5–3 years; 5: 3–5 years; 6: 5–7 years; 7: 14–22 years; 8: 39–79 years; for details, consult Table 1. For CPS and ASS, expression in groups 1–4 was significantly higher than in groups 5–8, whereas for OAT, expression in prenatal group1 was higher than in the postnatal groups (for details, see main text). Significant differences in expression between villus and crypt enterocytes are indicated by an asterisk if P ≤ 0.005, a "#" if P ≤ 0.017, and a "+" if P ≤ 0.04. Significant differences between age groups are described in the Results section.

**Figure 3:**
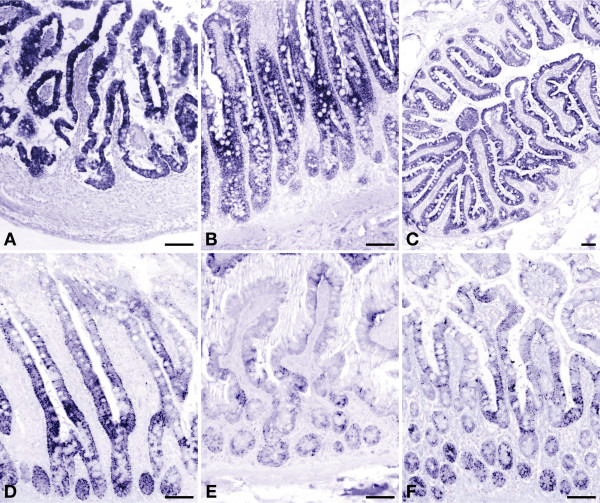
**Developmental changes in expression of carbamoylphosphate synthetase in human small intestine**. Panel A: gestational week 14; panel B: jejunum of a 3-day-old term male neonate; panel C: ileum of a 3-year-old male toddler; panel D: ileum of a 6-year-old male child; panel E: ileum of a 22-year-old female patient; panel F: ileum of a 70-year-old female patient. Scale bar: 100 μm.

CPS protein was exclusively found in the enterocytes. Prior to birth (group 1), CPS expression was uniformly high in the enterocytes of both crypts and villi (Figure 3A). Between birth and 3 years of age (groups 2–4), CPS expression did not change significantly in the enterocytes on the villi, but declined thereafter to reach adult levels by 5 years of age (difference in villous expression between groups 1–4 vs. groups 5–8: P ≤ 0.0001). Between birth and 1 year of age, expression was higher in the villus than in the crypt enterocytes (P < 0.005; Figure 3B [see Additional file 2]). Thereafter, staining differences between villi and crypts disappeared again (Figure 3C–F). No expression of CPS was found in the epithelium of Brunner's glands [see Additional file 3].

#### Ornithine aminotransferase (Figures 2B and 4; [see also Additional files 2 and 3])

**Figure 4:**
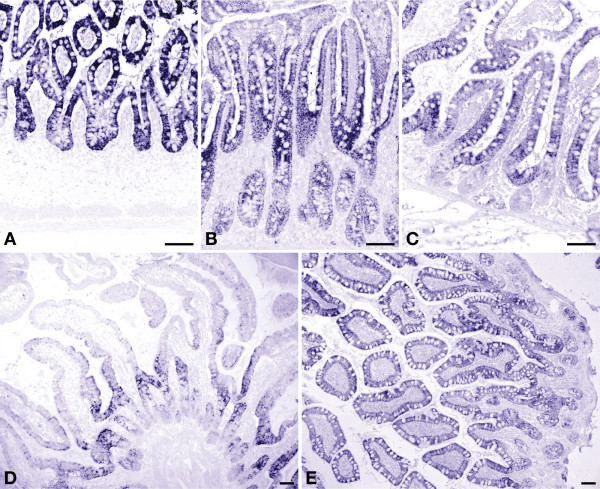
**Developmental changes in expression of ornithine aminotransferase in human small intestine**. Panel A: gestational week 23; panel B: jejunum of a 3-day-old term male neonate; panel C: ileum of a 3-year-old male toddler; panel D: ileum of a 6-year-old male child; panel E: ileum of a 70-year-old female patient. Scale bar: 100 μm.

OAT expression in the epithelium was uniform prior to birth. The youngest samples that could be investigated were from gestational week 23 (Figure 4A). Fetal expression was significantly higher in both crypts and villi than in the other age groups (P ≤ 0.004). Directly after birth, OAT expression remained strong in the epithelium covering the villi, but declined in the crypts (P ≤ 0.017), with the exception of age group 4 (1, 5-3 years). In some preparations, expression was stronger at the base of the villi, just above the crypts (Figure 4B, D). Above 40 years of age, OAT expression again resembled the fetal pattern, with crypt and villus enterocytes expressing OAT almost evenly albeit at a lower level (Figure 4E). The epithelium of Brunner's glands did not express OAT [see Additional file 3].

#### Argininosuccinate synthetase (Figures 2C and 5; [see also Additional files 2 and 3])

**Figure 5:**
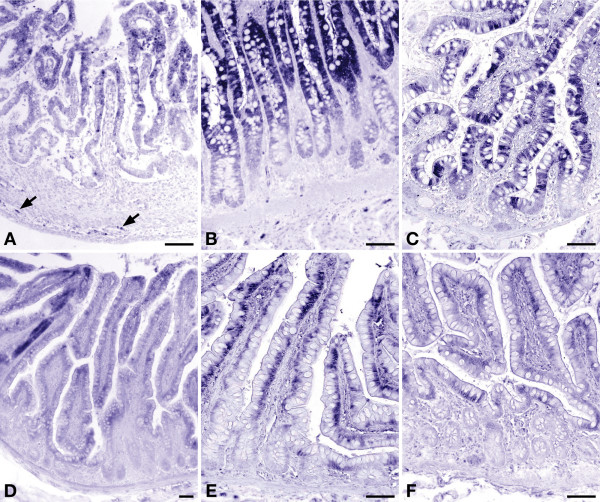
**Developmental changes in expression of argininosuccinate synthetase in human small intestine**. Panel A: gestational week 14; panel B: jejunum of a 3-day-old term male neonate; panel C: ileum of a 3-year-old male toddler; panel D: ileum of a 6-year-old male child; panel E: ileum of a 22-year-old female patient; panel F: ileum of a 70-year-old female patient. Arrows in panel A indicate ASS-positive neurons in the myenteric plexus. Scale bar: 100 μm.

With the exception of the age groups 5 and 6 (3–7 years), ASS protein accumulated to a much higher concentration in the enterocytes on the villi than in the crypts (P < 0.014), especially before 3 years of age (P < 0.004). This pattern was already found in gestational week 14, where epithelial expression of ASS was still low (Figure 5A). From week 23 onward, expression in the villus epithelium was intermediate to strong. ASS expression remained high during the first postnatal year (groups 2 and 3 vs. groups 5–8, P < 0.0001), and then declined via an intermediate score between 1.5 and 3 years (group 4) to a near-absent score between 3 and 5 years (group 5). ASS expression in the crypts slowly declined after birth to become undetectable after 3 years of age (Figure 5B–F; [see also Additional file 2]) (Groups 1–4 vs. groups 5–8 in a multiple comparison of groups: P < 0.0001). As long as ASS expression was high (i.e. in the first 3 years), ASS protein was present throughout the enterocytes (Figure 5A–C), but in children and young adults, it gradually became concentrated at the basal side of the enterocytes (Figure 5D–F). No expression of ASS was found in the epithelium of Brunner's glands [see Additional file 3].

#### Arginase-1 (Figure 6A)

**Figure 6:**
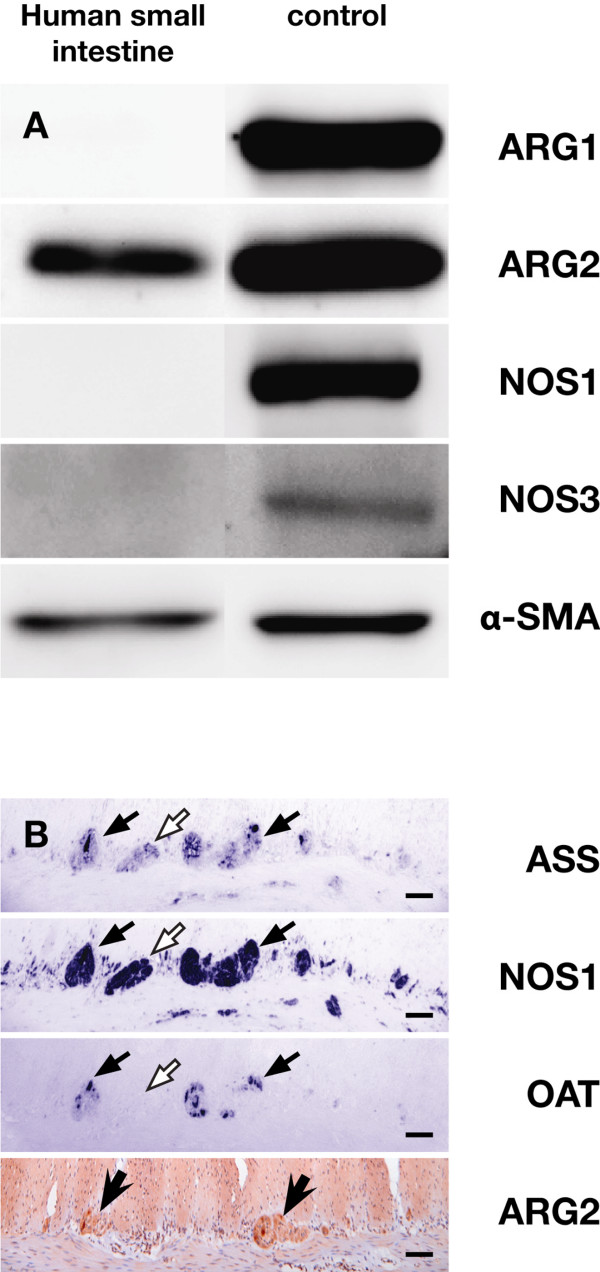
**Expression of ASS, ARG and NOS in villus epithelium and neurons**. Panel A: 40 μg of protein isolated from small-intestinal scrapings of a 60-year-old patient were loaded per lane (left column). Positive controls (right column) included 25 μg of human liver extract for ARG1, NOS1 and α-SMA, 25 μg of human kidney extract for ARG2, and 25 μg of mouse brain extract for NOS3. Absence of ARG1, NOS1 and NOS3 expression in the adult small-intestinal enterocytes is demonstrated. The density of the ARG2 band is approx. 10% of that in kidney. Panel B: Serial sections (5 μm) of the ileal myenteric plexus of a 6-year-old male child were stained for ASS, NOS1, and OAT. Note colocalization of ASS, NOS1, and OAT in intensely staining neurons (black arrows). Also note OAT-negative ganglia (white arrows). Another section of the same specimen was stained for ARG2. Scale bar: 100 μm.

ARG1, the major arginine-catabolizing enzyme, was not detectable immunohistochemically in the epithelium of the small intestine at any of the ages investigated. Western-blot analysis of samples from an adult duodenum (age: 60 years) were also negative for ARG1, confirming the immunohistochemical findings.

#### Arginase-2 (Figures 2D, 6A, and 7)

**Figure 7:**
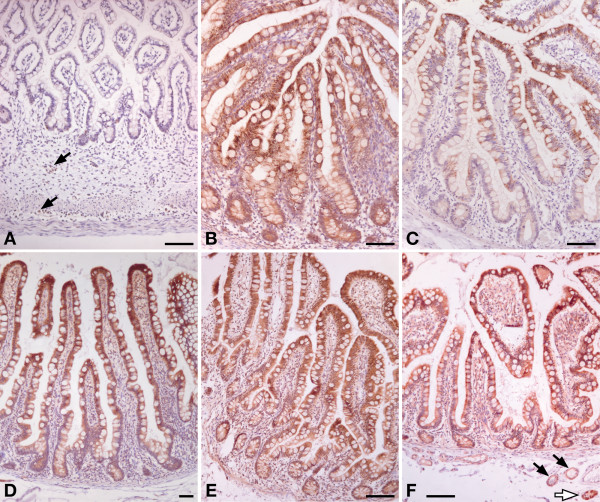
**Developmental changes in expression of arginase-2 in human small intestine**. Panel A: gestational week 23; arrows indicate positive neurons in submucosal and myenteric plexus; panel B: jejunum of a 3-day-old term male neonate; panel C: ileum of a 3-year-old male toddler; panel D: ileum of a 6-year-old male child; panel E: ileum of a 22-year-old female patient; panel F: ileum of a 70-year-old female patient; the white arrow shows staining of a submucosal ganglion, the black arrows indicate staining of smooth muscle cells in the wall of arterioles. Scale bar: 100 μm.

Western blots of the adult duodenum showed, instead, expression of the mitochondrial isoform of arginase (Figure 6A). We therefore investigated expression of ARG2 in a subset of samples (2–4/group) from all age-groups analyzed for ASS, CPS and OAT expression. ARG2 expression in fetal enterocytes was very weak (Figure 7A) or absent, but in neonates and all older age groups, expression was significantly higher (P < 0.02). After birth, protein expression in enterocytes was stronger on the villi than in the crypts (Figure 7B–F; P < 0.0001 for all groups in a multiple comparison; due to the relatively small sample size this difference was not significant for all individual groups).

#### NOS1 (Figure 6A)

Over-staining of sections with NOS1 yielded weak epithelial staining. Enterocytes of the human small intestine express high levels of NOS1 mRNA, but protein was not detected [33]. In agreement, a Western blot of a protein extract from villus epithelium did not show staining for NOS1, demonstrating that the observed staining of the sections was due to non-specific antibody binding.

#### NOS3 (Figure 6A)

We found weakly positive staining of villus epithelium with our NOS3 antibody. NOS3 mRNA is not expressed in enterocytes [33]. We, therefore, incubated a Western blot of a protein extract from epithelial cells with the same antibody as used for immunohistochemistry, but no immunoreactivity with NOS3 could be demonstrated, showing that the observed staining of the sections was due to non-specific antibody binding.

#### Localization of enzymes involved in arginine biosynthesis (Figure 8)

**Figure 8:**
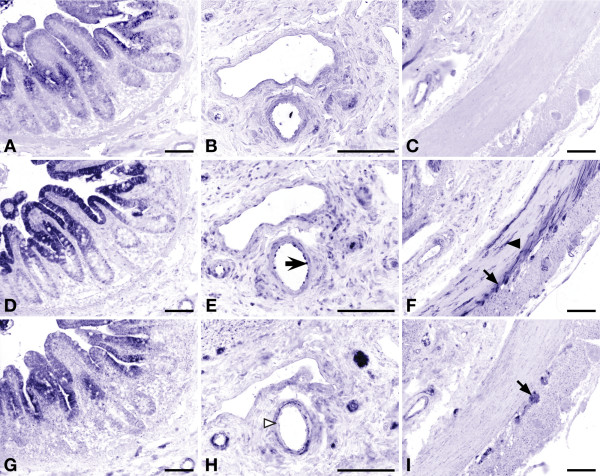
**Expression of enzymes involved in arginine synthesis in human small intestine at postnatal day 1**. Serial sections of the distal duodenum of a 1-day-old female neonate. Panels A-C show the expression of CPS; panels D-F: ASS; and panels G-I: OAT. Endothelial expression of ASS in panel E is indicated by block arrows, while OAT expression in the smooth muscle layer of the same vessels is indicated by a white arrowhead (panel H). Panels C, F and I show the muscularis propria with the inner circular and outer longitudinal layer. Myenteric ganglia are indicated by black arrows in panels F and I. Varicose nerves in the circular muscle layer are indicated by an arrowhead in panel F. Scale bar: 100 μm.

Serial sections of the distal duodenum at postnatal day 1 demonstrated that the highest expression of CPS, ASS, and OAT was found in the enterocytes covering the villi (Figure 8A, D, G). CPS-, OAT-, and ASS-positive enterocytes did not express ARG1 (not shown). The positive staining of the ARG1-rich lysed erythrocytes [34] inside vessels served as an internal positive control for the absence of ARG1 staining in enterocytes. ASS was also expressed in the endothelium of the small arteries (Figure 8E, arrow), while OAT was present in the smooth muscle wall of these vessels (Figure 8H, white arrowhead). In addition, ASS and OAT were expressed in the ganglia of the myenteric plexus (Figure 8F, I, arrows). Only ASS was prominent in the varicose nerves of the circular muscle layer (Figure 8F, arrow head) and, to a lesser extent, in those of the longitudinal muscle layer.

#### Expression of enzymes in enteric nerves (Figure 6B)

Single neurons in the ganglia of both the myenteric and the submucosal plexus stained very strongly for ASS, OAT, ARG2, and NOS1, whereas other neurons in the same ganglion stained weaker or not at all. The strongly OAT-positive neurons were also positive for ASS and NOS1 (Figure 6B, black arrows), but some ganglia that were positive for both NOS1 and ASS did not express OAT (Figure 6B, white arrows). ASS- and NOS1-negative neuronal bodies were not observed in ganglia. Considerable staining of varicose nerve fibers for ASS and NOS1, but not for OAT, was observed in the circular and, to a lesser extent, the longitudinal smooth-muscle layer (Figures 6B and 8F). Positive staining for ASS of single neurons in myenteric ganglia was already found in gestational week 14 (Figure 5A, arrows), whereas NOS1 (data not shown) and ARG2 were detectable in gestational week 23 (Figure 7A, arrows). ASS, OAT, and ARG2 expression was also found in ganglia of the outer and inner submucosal plexus (Figures 6B and 7F). The neurons of the myenteric plexus stained stronger than those of the submucosal plexus, except those positive for ARG2, which stained equally strong in both.

#### Expression of enzymes in the wall of intestinal vessels

The endothelium of the small arterioles in the submucosa and serosa always stained positive for the presence of ASS (Figure 8E, arrow) and NOS3, but that of the larger arteries, veins and lymph vessels was negative for both enzymes. After birth, OAT and ARG2 expression was demonstrable in the smooth-muscle cell layer of arterioles (Figures 7F). No expression was found in the smooth-muscle cell layer of veins.

#### Expression of enzymes elsewhere in the intestine

Single cells in the lamina propria that were strongly positive for NOS1 were also positive for CD68, a marker for macrophages, but not for CD3, CD20, CD138, or CD1A (lymphocytes and dendritic cells (not shown). Germinal centers within lymphocyte aggregates also stained positive for NOS1 (not shown).

## Discussion

The main result of this study is the observation that the epithelium of the perinatal human small intestine expresses the enzymes that exert control over the biosynthesis of arginine from proline, bicarbonate, and ammonia, viz. CPS, OAT and ASS (See Figure 1), and that the major arginine-degrading enzyme ARG1 is absent during that period. We deduced this conclusion from an enzyme-histochemical analysis of 79 specimens less than 8 years old.

### The human small intestine expresses key enzymes of arginine synthesis at midgestation

Although the developmental appearance of CPS, OAT, and ASS in the enterocytes of the piglet and rodent small intestine has been reported [6-9,35-38], such information was only incompletely available for the human small intestine. CPS expression in the human small intestine starts as early as the 8^th ^week of gestation [23,24]. Accordingly, CPS was expressed in the enterocytes of all our samples. To our knowledge, OAT expression in human enterocytes has not yet been reported, but its developmental profile resembles that of CPS, ornithine carbamoyltransferase and pyrroline-5-carboxylate reductase in fetal piglets [37]. In agreement, OAT was, like CPS, expressed in the enterocytes of all our samples. ASS, finally, is expressed in Caco2 cells [39], but its expression in normal human small intestinal enterocytes has not yet been reported. ASS differed from CPS and OAT in that its expression declined profoundly between 3 and 5 years of age (Figure 2). The time course in ASS expression in the postnatal human gut resembles that in piglets, which declines towards weaning and then rises again [10]. In rodents, on the other hand, ASS expression disappears completely at weaning [6,40]. Concurrent with the developmental decline in ASS expression, its homogeneous cytosolic distribution changed to one that is restricted to the basal side of the enterocytes (Figure 5E, F). Asymmetric localization of proteins in enterocytes has been previously described as a consequence of mRNA sorting [41], but a change in enzyme localization during late postnatal development has, to our knowledge, not yet been demonstrated.

The crypt-villus gradient of postnatal CPS and ASS expression in the suckling human intestine resembles that in piglets (our unpublished observations), but markedly differs from that in rodents. In both rats and mice [5,6], ASS expression is confined to the enterocytes occupying the apical half of the villi, whereas CPS is expressed in all enterocytes. This expression pattern suggested to us that the basal enterocytes synthesized citrulline, whereas the apical enterocytes synthesized arginine [6]. A possible explanation for this spatial separation of cells capable to produce citrulline only and cells also capable of arginine production is that the small-intestinal enterocytes are the only cells in the body that can synthesize citrulline and that this citrulline is necessary as substrate for arginine synthesis elsewhere, e.g. the kidney and the endothelium. Apparently, such a zonation of arginine metabolism is not necessary in newborn pigs and humans.

The net production of ornithine by the small intestine is a prerequisite for citrulline and arginine biosynthesis [42]. The severe deficiency of circulating ornithine and arginine that develops in neonates lacking OAT [38,43] demonstrates that, in the neonatal intestine, OAT indeed serves to produce ornithine. Citrulline is exported from the mitochondria in exchange for ornithine via the ornithine carrier ORNT. Accordingly, the ORNT1 isoform is expressed at elevated levels in the small intestine of the mouse during the suckling period [44]. However, the relatively high expression of ARG2, the mitochondrial isoform of arginase, in the neonatal and adult human small intestine could potentially nullify the cytosolic synthesis of arginine (in the mouse small intestine, ARG2 becomes only expressed at weaning [5,6]). Extensive degradation of cytosolic arginine due to import into the mitochondria is, nevertheless, unlikely, because the affinity of arginine for the ORNT carrier is ~10-fold lower than that of ornithine [45].

Together, our findings indicate that the human fetal intestine has acquired the potential to produce arginine at 23 weeks of gestation and probably as early as 14 weeks, that is, well before fetuses become viable outside the uterus. Furthermore, NOS1, NOS3 and their downstream target soluble guanylate cyclase (not shown) were expressed at term levels in fetuses of ~23 weeks of pregnancy. This indicates that at this time in gestation the fetus not only has the potential to synthesize arginine, but also to use it for NO and cyclic GMP production. Hypoargininemia, nevertheless, often develops in preterm infants, in particular if they are maintained on total parenteral nutrition [5,6,15], and appears to predispose them to the respiratory distress syndrome [46] and necrotizing enterocolitis [19,20]. The hyperammonemia that frequently accompanies hypoargininemia in preterms responds to intravenous arginine supplementation [18], which indicates that endogenous arginine biosynthesis is deficient. The strong association with parenteral nutrition points to the intestines and indicates that the intestines only produce arginine if substrate is supplied from the intestinal lumen, as was shown for the newborn piglet [11,12].

### Intestinal neurons and arterioles express key enzymes of arginine synthesis

The neurons of the myenteric plexus abundantly express OAT, ASS, and NOS1. Since the neurons do not express CPS and, therefore, cannot synthesize citrulline from ornithine, OAT most likely functions to produce glutamate, while ASS probably functions to (re-)synthesize arginine from citrulline [47]. It is conceivable that these neurons require under certain conditions additional arginine (synthesized by enterocytes) above their endogenous production. Such metabolic cooperation has been demonstrated for the intestinal sphincters: neurons in these sphincters can resynthesize arginine from citrulline, but become dependent on external arginine during prolonged activity [48]. The expression pattern of soluble guanylate cyclase (not shown) demonstrated that there are many target cells for the NO produced by the neurons of the myenteric plexus. In this respect, the inner, circular layer of the muscularis propria differed markedly from the outer, longitudinal layer both by the far more abundant distribution of ASS- and NOS1-positive nerve fibres and its much stronger expression of soluble guanylate cyclase (not shown). The much stronger immunoreactivity for NOS1 of the nerve fibers in the circular muscle has been described [49]. Although nerve cell content and density of the NOS-positive ganglia of the myenteric plexus [49-51] markedly declines in the peri- and postnatal period, this structural difference between both layers of the muscularis propria is maintained throughout development.

The constantly fed state of the neonatal small intestine causes absorptive hyperemia, that is, a high intestinal blood flow and a low intestinal vessel resistance [52]. NO production in the vessel wall is the main determinant of this vascular relaxation [53], in particular during the suckling period [54]. Since neonatal hypoargininemia is closely associated with the risk to develop necrotizing enterocolitis, a disease of the intestinal vessels [55], we speculate that arginine biosynthesis in both the enterocytes and the endothelial cells of the small vessels of the intestinal submucosa is necessary to support this hyperemic state and to protect the neonatal intestine from ischemia. Unfortunately, only one intervention trial [56] without a conclusive outcome [57] has tested the predicted beneficial effect of arginine supplementation to preterm neonates thus far. Although arteries of the 1^st ^to 2^nd ^order (out of 4 size categories) are considered to be the major sites of resistance and blood flow regulation of the gut [58], we did not observe ASS and NOS3 expression in the endothelium of 1^st ^order (largest) arteries. Furthermore, the downstream target of NO, soluble guanylate cyclase, was found in the smooth-muscle layer of arterioles only (not shown), suggesting that these 2^nd ^order vessels materially contribute to the peripheral vascular resistance in the gut. A recent study showed a deficiency in NO production but not in NOS3 expression as judged by immunohistochemistry of intestinal arterioles of patients with NEC [59], supporting our hypothesis that NOS substrate deficiency plays a role in intestinal ischemia. In addition to NO production, other factors are involved in neonatal vascular dysfunction, such as the pro-inflammatory cytokine IL-1β and endothelin-1 [60], but their effects are mediated at least in part via a blunting of NOS3-mediated NO production.

### The decline of arginine-synthesizing enzymes in enterocytes and "weaning"

The age-dependent postnatal decline in the expression of ASS in the enterocytes of the human small intestine is most pronounced between 3 and 5 years of age. The time course in ASS expression in the developing human gut resembles that in piglets, where activity is highest during the suckling period, declines to low levels around weaning (under natural conditions occurring at 12–15 weeks [61]) and then rises again [10]. In rodents, on the other hand, ASS expression disappears completely at weaning [5,6,15]. In humans, approximately 6 months of age is considered to be an optimal time point for weaning, but the natural weaning age for humans may be as late as 2.5–3 years [25]. The prominent reduction of ASS expression that we observed after three years of age, therefore, coincides with this assumed natural weaning age.

There is a remarkable similarity in the developmental timing of the decline in expression of ASS and lactase-phlorizin hydrolase (hereafter called lactase), another small-intestinal enzyme that is closely associated with breast-feeding. In rodents, lactase expression declines to undetectable levels at weaning [62], whereas in piglets, the decline occurs more gradually during the first 8–16 weeks of life [63]. In lactase-nonpersistent humans, lactase activity begins to decline between 2 and 3 years [64]. The temporal coincidence of the intestinal capacity to digest lactose and to produce arginine does support a relation to milk as the main source of food and underscores the notion that mammalian milk does not contain enough arginine to support rapid postnatal growth [17], so that intestinal arginine synthesis is necessary. Although we did find low levels of ASS in adult intestine, it was recently reported that the human intestine (age range 37–69 years) does not produce arginine [65]. This finding does not exclude a role for local arginine synthesis in e.g. neurons and endothelial cells that is directly coupled to NO production within the same cell.

## Conclusion

Our data show that CPS, ASS, and OAT expression is strong in the enterocytes of fetuses, neonates, infants and toddlers. Humans, therefore, resemble other mammals in that the enterocytes of their small intestine are major producers of arginine during the suckling period. Although the relative deficiency of arginine in milk seems to underlie this temporary function of the gut, the intestinal or systemic functions that require the arginine that is produced in the gut remain to be delineated. We submit that relaxation of the circular smooth muscle layer of the muscularis propria [49,66] and that of the intestinal arterioles [54] stand out in this respect.

## Abbreviations

ARG: arginase (E.C. 3.5.3.1); ASS: argininosuccinate synthetase (E.C. 6.3.4.5); CPS: carbamoylphosphate synthetase (E.C. 6.3.4.16); NOS: nitric oxide synthase (E.C. 1.14.13.39); OAT: ornithine aminotransferase (E.C. 2.6.1.13).

## Authors' contributions

EK participated in the design of the study and carried out the data analysis and drafted the manuscript. SS assisted in data analysis and processing. CG assisted in the study design, data analysis and critically revised the manuscript. PD carried out immunoassays. JV carried out immunoassays. JR assisted in the design of data analysis and performed the statistical analysis. WL conceived of the study, participated in analysis and interpretation, and critically revised the manuscript for important intellectual content. EB is the pediatric pathologist of this study, was responsible for the diagnoses and data interpretation and critically revised the manuscript.

## Supplementary Material

Additional file 1 **Representative negative controls.** Click here for file

Additional file 2 **Expression of ASS, CPS and OAT in a group 3 patient.** Click here for file

Additional file 3 **Brunner glands in a group 5 patient.** Click here for file
