# Supplementary material for: The human neonatal small intestine has the potential for arginine synthesis; developmental changes in the expression of arginine-synthesizing and -catabolizing enzymes
Source: BMC Dev Biol. 2008 Nov 10;8:107. doi: 10.1186/1471-213X-8-107 (PMC2621195; doi:10.1186/1471-213X-8-107)
Supplement: Additional file 1 — Representative negative controls. [file 1471-213X-8-107-S1.pdf]

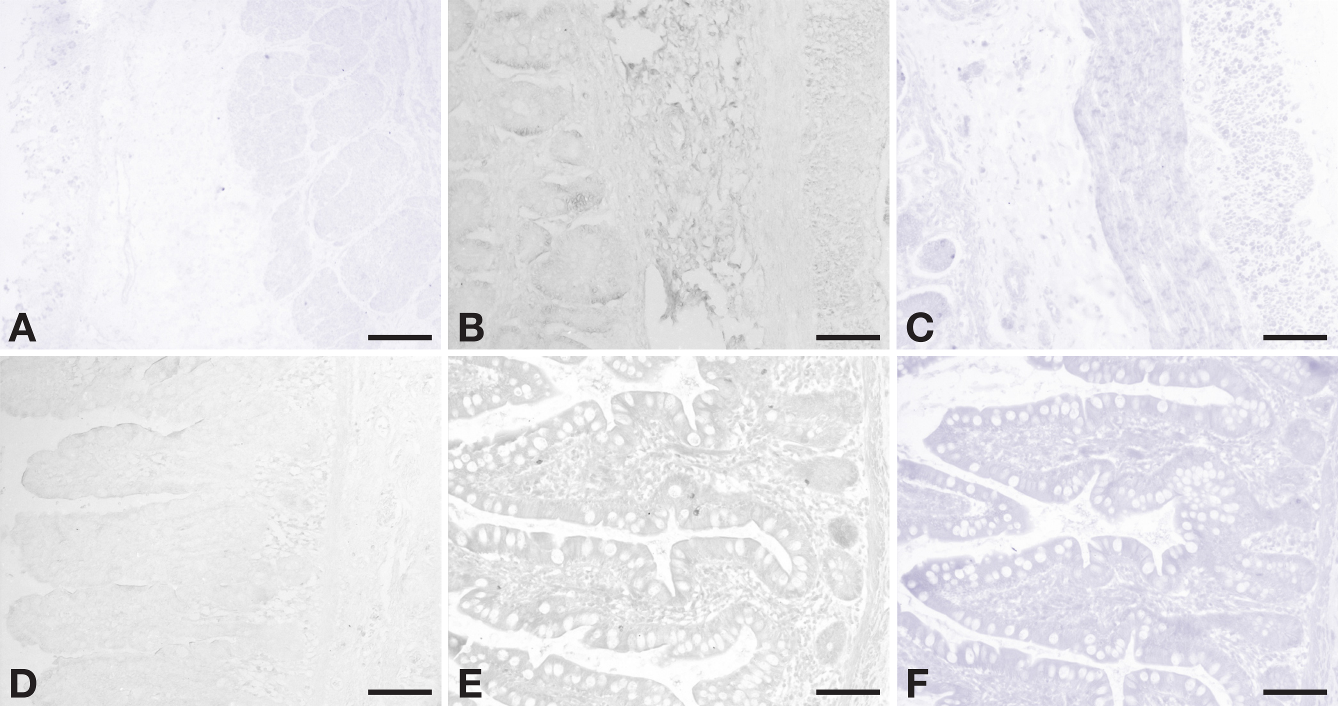

Representative negative controls
A) Fetal sample (alkaline phosphatase-coupled secondary antibody)
B) Sample of a 30-month-old patient (peroxidase-coupled sec. antibody)
C) Sample of a 30-month-old patient (alkaline phosphatase-coupled sec. antibody
D) Sample of a 1-day-old patient (peroxidase-coupled sec. antibody)
E) Sample of a 75-month-old patient (peroxidase-coupled sec. antibody)
F) Sample of a 75-month-old patient (alkaline phosphatase-coupled sec. antibody)
Scale bar 100 μm.
