# Supplementary material for: The human neonatal small intestine has the potential for arginine synthesis; developmental changes in the expression of arginine-synthesizing and -catabolizing enzymes
Source: BMC Dev Biol. 2008 Nov 10;8:107. doi: 10.1186/1471-213X-8-107 (PMC2621195; doi:10.1186/1471-213X-8-107)
Supplement: Additional file 2 — Expression of ASS, CPS and OAT in a group 3 patient. [file 1471-213X-8-107-S3.pdf]

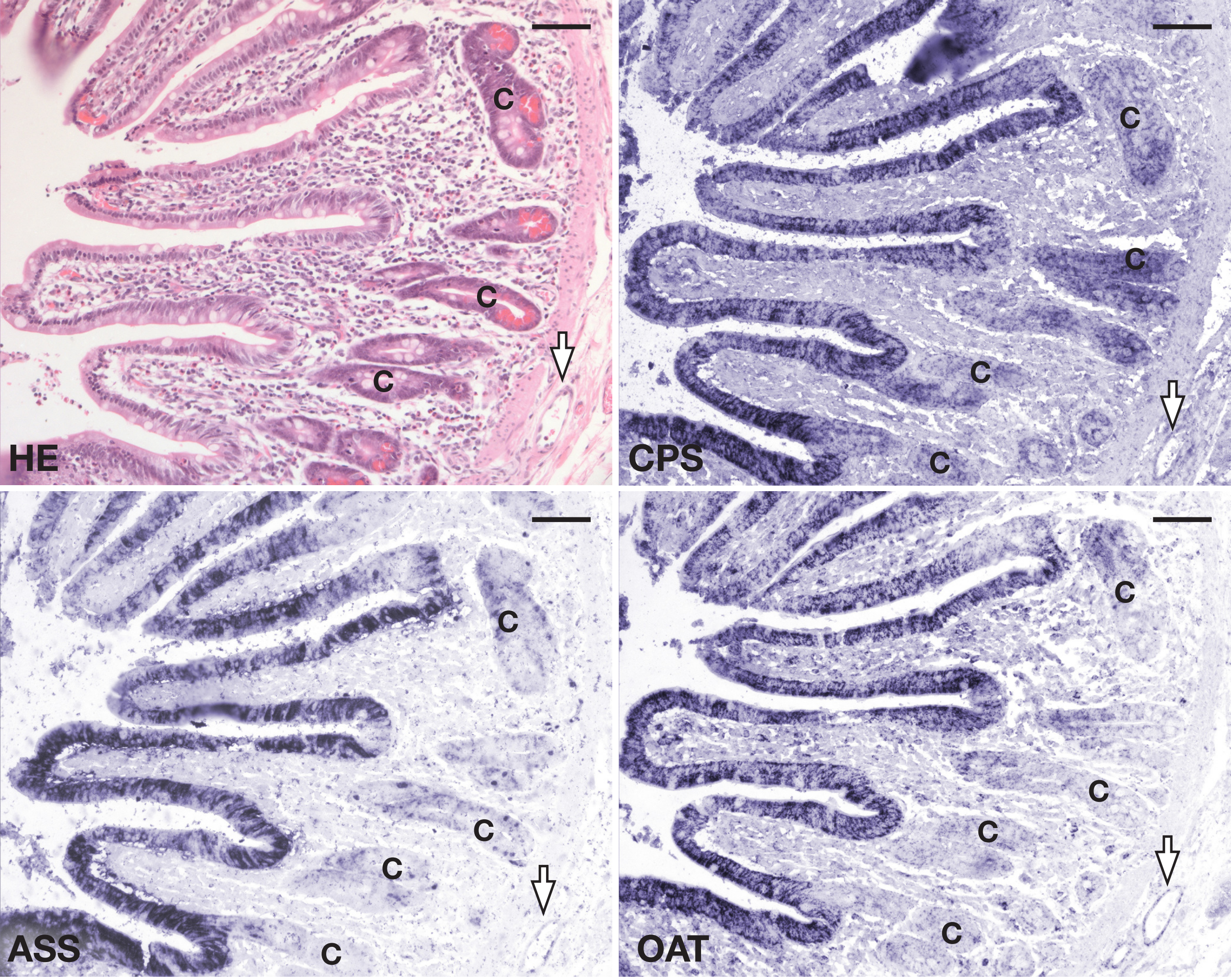

Expression of ASS, CPS and OAT in a group 3 patient
Serial sections of the duodenum of an 8-month-old male patient.
Top left: H&E staining; top right: CPS; bottom left: ASS; bottom right: OAT. All three enzymes stain stronger in the villi than in the crypts (C). An arteriole is indicated by an arrow –endothelial staining of ASS and smooth muscle cell staining of OAT are visible in the wall of this vessel. Scale bar: 100 µm.
