# Supplementary material for: The human neonatal small intestine has the potential for arginine synthesis; developmental changes in the expression of arginine-synthesizing and -catabolizing enzymes
Source: BMC Dev Biol. 2008 Nov 10;8:107. doi: 10.1186/1471-213X-8-107 (PMC2621195; doi:10.1186/1471-213X-8-107)
Supplement: Additional file 3 — Brunner glands in a group 5 patient. [file 1471-213X-8-107-S2.pdf]

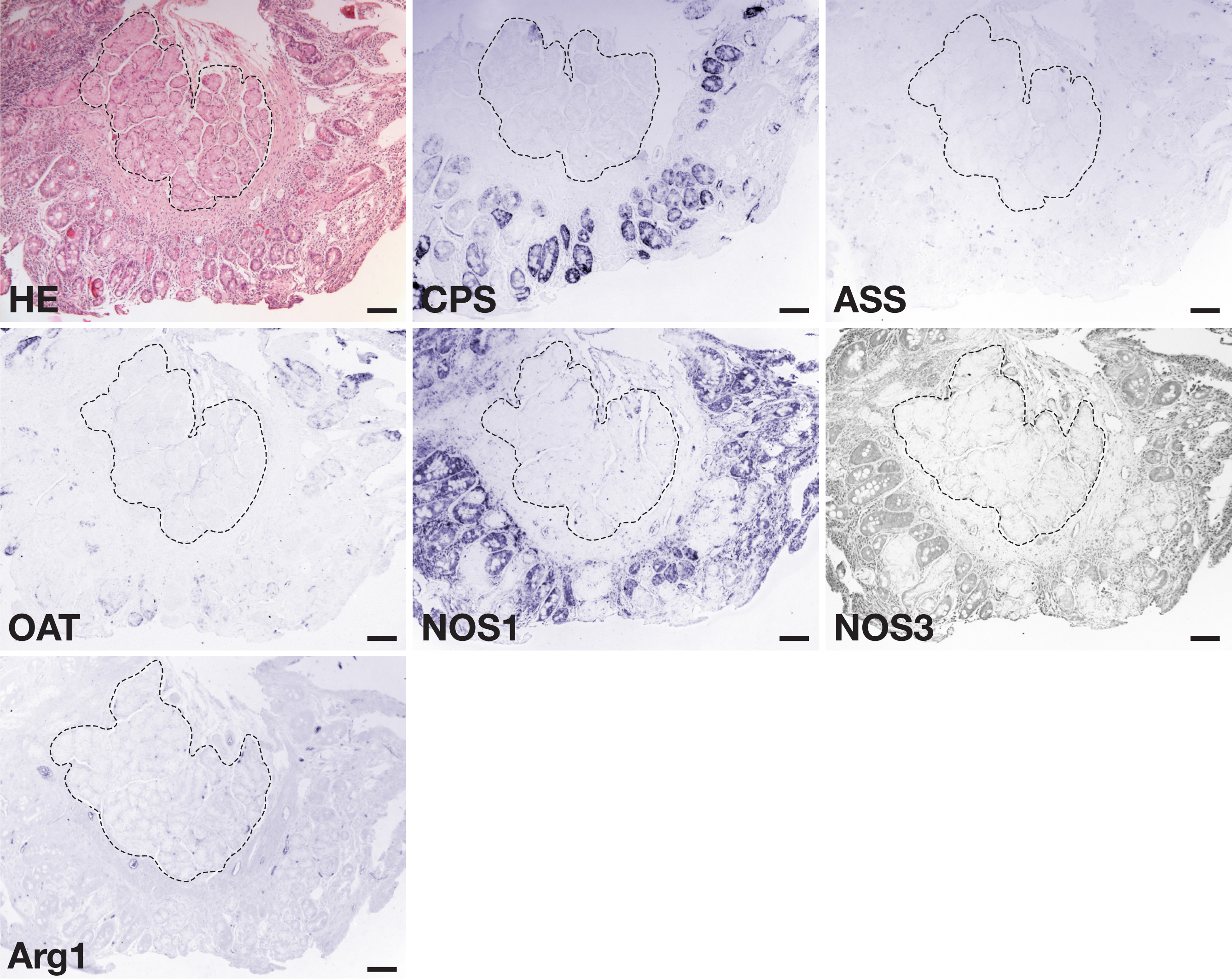

Brunner glands in a group 5 patient
Serial sections of a duodenum biopsy of a 40-months-old male patient. The Brunner glands are circled by a dotted line. The surrounding tissue contains only crypts. H&E and the different enzymes stained are indicated per picture. Scale bar 100 µm.
